# Desmoplastic small round cell tumour in the Afro-Caribbean population: case report with imaging findings

**DOI:** 10.1259/bjrcr.20190060

**Published:** 2020-02-12

**Authors:** Kino Ceon Francis, Chevar O’Shay South, Bonnie-Paul Regis Williams, Kurt Garey Gabriel, Mahiri Bromfield

**Affiliations:** 1 Department of Diagnostic and Interventional Radiology, University Hospital of the West Indies, Mona, Kingston, Jamaica; 2 Department of Pathology, University of the West Indies, Mona, Kingston, Jamaica

## Abstract

Desmoplastic Small Round Cell Tumour (DSRCT) is a rare malignancy that has only a few cases documented in the literature. We report a case of DSRCT in the abdomen and pelvis that was identified following ultrasound-guided biopsy of one of the numerous liver lesions seen on imaging in a 13-year-old Afro-Caribbean female with increased abdominal girth. The tumour was characterized by all routine imaging modalities available at the time. To our knowledge, this is the first reported and published case in the English speaking Caribbean. In the review of the literature, we correlate the imaging findings with previously reported cases. The diagnosis of DSRCT cannot be made solely using standard imaging techniques, but radiologists should be suspicious of DSRCT as a differential diagnosis in a young patient with increased abdominal girth, multiple liver and peritoneal deposits seen on imaging. Written informed consent for the case to be published (incl. images, case history, and data) was obtained from the parents of this patient for publication of this case report, including accompanying images.

## Case presentation

This is the case of a 13-year-old female known to have asthma since early childhood with her last attack being approximately 1 year ago. She was on maintenance medication of beclomethasone 50 mcg, and Salbutamol inhaler, two puffs as needed. She was non-compliant with her medication citing lack of symptoms as her reason for non-compliance. She presented with complaints of generalized weakness and fatigue for the past 6 days, along with weightloss of an uncertain number over the previous months. The patient noted that her clothing began fitting more loosely, particularly over the past 7 days. She reported no history of fever, joint or muscle pain, rashes, but stated that she was experiencing constipation, loss of appetite, but no nausea, vomiting, or any abdominal pain. Additionally, she reported early satiety; only being able to tolerate approximately two spoonfuls of food before she felt full. This was also associated with increased abdominal girth which was noted during the previous months. There was no reported yellowing of the eyes, dark urine or pale stool. Menarche was reported to be at age 12, with regular periods that lasted approximately for 5 days, requiring 3–4 changes of partially soiled pads per day. There was no history of sexual contact, intravenous drug use, or blood transfusions. She also reported having significant exercise intolerance; only being able to walk about 20.0 m before she became tired. There were no palpitations or chest pain experienced but has shortness of breath on exertion. No urinary symptoms or history of seizure activities were reported. There was no other significant finding in her medical history. She presented to the hospital for further management. Pertinent examination findings were limited to the abdominal examination where our patient was noted to have mild epigastric prominence with a hypopigmented patch noted to the central abdomen. No caput-medusae, angiomas, hernias or pulsatile masses were appreciated. The abdomen was soft but tender in the right upper quadrant on deep palpation. A negative Murphy’s sign was appreciated. The liver span measured 24.0 cm, with no splenomegaly appreciated. The kidneys were not ballotable and there was no renal angle tenderness. There was no shifting dullness or transmitted fluid thrill appreciated. Notably, the patient was underweight with a calculated body mass index of 17.6. A clinical assessment of tender hepatomegaly of unknown cause was made, to rule out a primary versus a secondary hepatic mass.

## Investigation

A complete blood count, urea and electrolytes, coagulation profile (prothrombin and partial thromboplastin time), hepatitis B, hepatitis C, and liver function tests were ordered along with an abdominal ultrasound, urinalysis, and urine beta-human chorionic gonadotropin (HCG). The urine β HCG was negative, the liver function tests were normal except for elevated alkaline phosphatase and gamma-glutamyl transferase. The complete blood count, the urea and electrolytes, as well as the coagulation profile, were within normal limits. The urinalysis was also normal.

**Figure 1.**
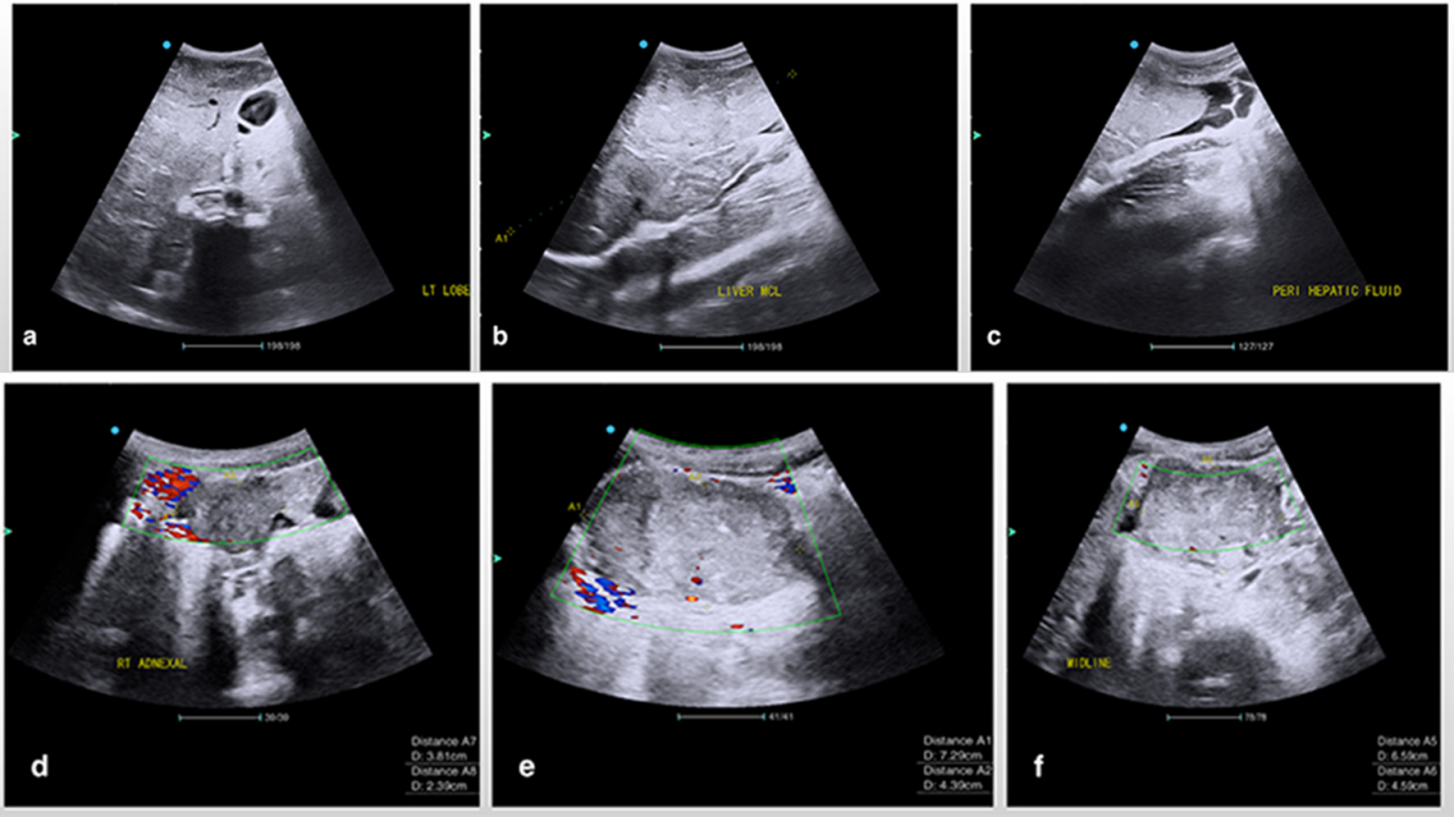
Abdominal ultrasound images in the sagittal and axial planes demonstrate the left and right lobes of the liver (b) with multiple isoechoic masses of varying sizes. Perihepatic free fluid consistent with ascites was also present (c) along with multiple heterogeneous hypovascular masses in the right adnexa (d), pelvis (e) and midline (f) regions.

The abdominal ultrasound revealed an enlarged coarse appearing liver (24.2 cm in span), with multiple ill-defined isoechoic masses of varying sizes along with anechoic perihepatic free fluid (Figure 1).

**Figure 2.**
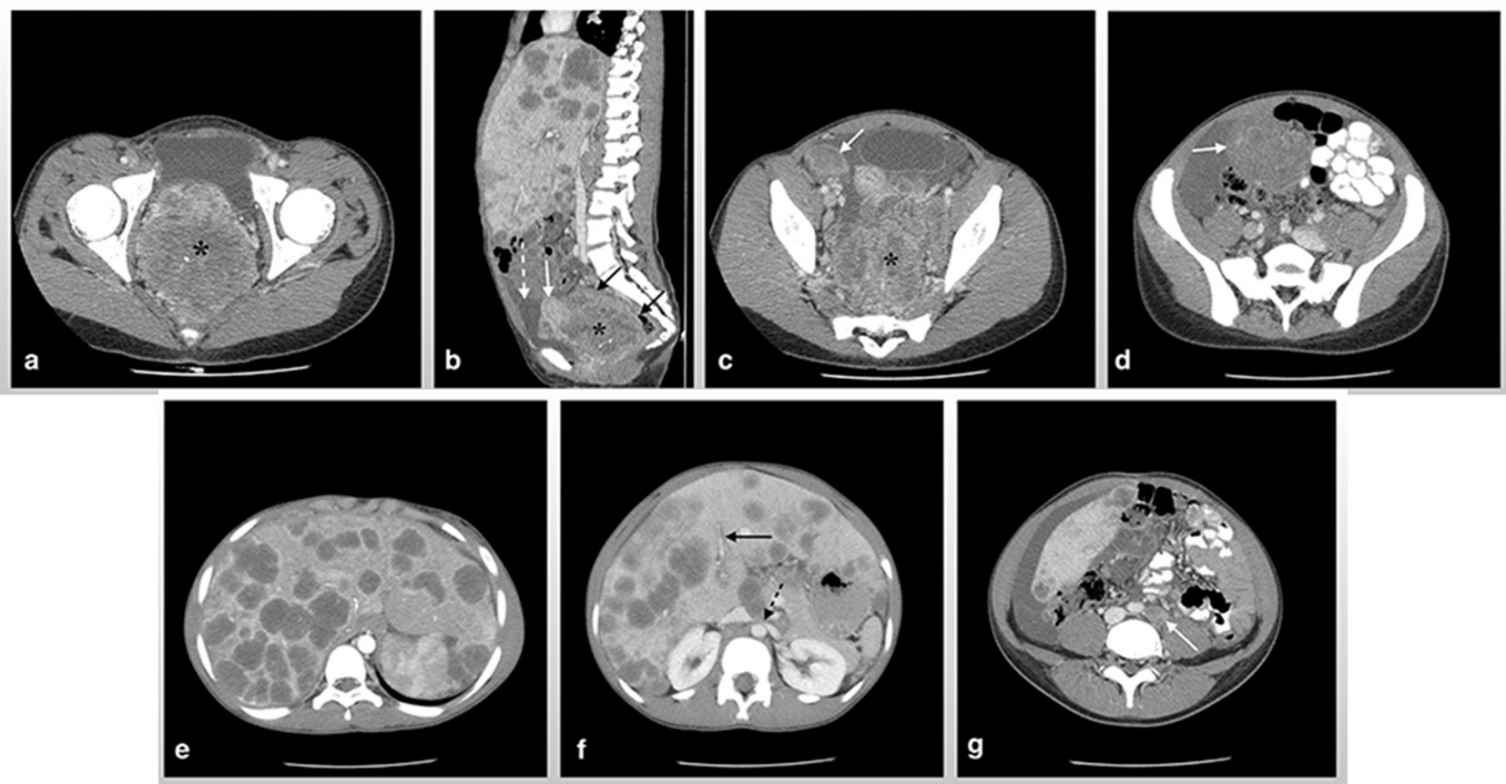
Axial CT scan image shows a large ill-defined heterogeneous solid enhancing mass (asterisk) in the pouch of Douglas within the central pelvis which demonstrates low attenuating central areas along with a few calcific foci (a). Sagittal CT scan image demonstrates mass effect on the uterus (white arrow) anteriorly without a plane of separation. The sigmoid rectum (black arrows) was collapsed and draped over the superior extent of the mass. It was also inseparable (b). Peritoneal deposits (white arrow) are seen in the right adnexa (c) and within the mesentery (d). Axial CT scan images show innumerable, predominantly rounded low attenuation (35.0 HU) masses (e), the intrahepatic portion of the inferior vena cava, hepatic veins (black arrow) and left renal vein (dotted black arrow) compressed (f) and para-aortic lymph nodes (white arrow) (g).

Multiple heterogeneous masses were also seen within the pelvis which appeared hypovascular on colour doppler interrogation. Moderate volume pelvic free fluid (ascites) was also visualized. These findings were highly suspicious for an underlying malignancy and the recommendation was made to have a CT scan of the abdomen and pelvis done. The CT scan of the abdomen and pelvis within the same admission period revealed a large ill-defined, heterogeneous solid mass with low attenuation central areas measuring 9.2 (L) x 9.4 (W) x 6.3 (AP) cm in the pouch of Douglas within the central pelvis (Figure 2).

Centrally, this lesion displayed a few subcentimetre foci of calcification. No intralesional fat density was seen within this mass. The mass demonstrated mass effect on the uterus anteriorly and a clear plane of separation was not seen. Posteriorly, the adjacent sigmoid rectum was collapsed and draped over the superior extent of the mass. It was also inseparable. The involvement of the latter structures could not be excluded. Normal appearing ovaries were not identified. Multiple similarly appearing solid masses consistent with extensive peritoneal deposits were seen in the right and left adnexa, posterior to the bladder, along the pelvis side-walls with the largest focus seen in the bowel mesentery measuring 6.8 × 5.1 cm. The liver was markedly enlarged (25.0 cm measured in the midclavicular line) and the left lobe extended into the left hypochondrium. The liver demonstrated innumerable, predominantly rounded low attenuation (35.0 Hounsfield unit) masses measuring from a few mm to a maximum of 6.5 × 5.6 cm; the largest lesion was seen in segment 7. There were patchy areas of heterogeneous hepatic enhancement. The intrahepatic portion of the inferior vena cava, hepatic veins, and the peripheral portal veins were compressed; however, blood flow was maintained. There was also left renal vein stenosis due to mass effect from an exophytic hepatic lesion.

Innumerable enlarged abnormal lymph nodes were also noted circumferentially encasing the celiac trunk, portal vein, aorta, common and internal iliac vessels. Mesenteric adenopathy was also seen. The constellation of findings on imaging was highly suspicious for malignancy and therefore the patient was referred for ultrasound-guided biopsy of the liver lesions, which was then sent for histopathology and immunohistochemistry (IHC).

**Figure 3.**
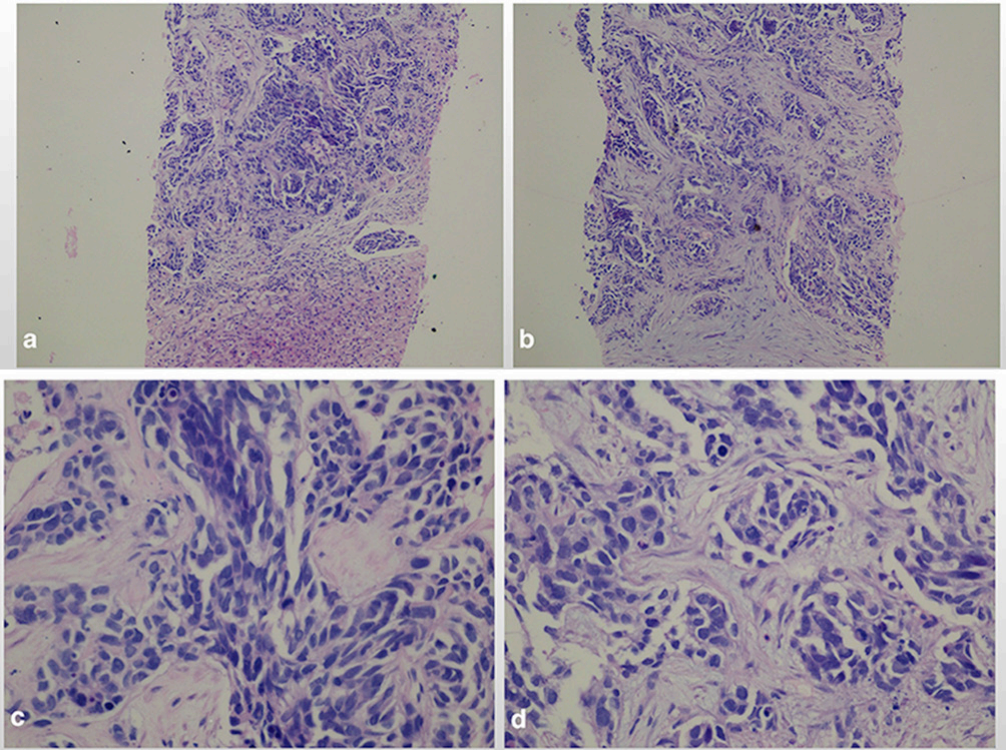
Sections showed cores of liver parenchyma extensively infiltrated by nests and trabeculae of malignant epithelioid cells with pale to eosinophilic cytoplasm and indistinct cell borders. The cell nuclei were basaloid and exhibited marked pleomorphism, hyperchromasia and brisk mitotic activity, including abnormal forms. Several areas of tumour necrosis were present and there was marked desmoplasia of the intervening stroma. Low power (x10) H&E (a), Low power (x 10) H&E (b), High power (x40) H&E (c) and High power (x40) H&E (d).

The biopsy consisted of three cores of tan to dark-brown tissue ranging in size from 1.5 × 0.1 × 0.1 cm to 1.0 x 0.1 × 0.1 cm (Figure 3).

The biopsy was sent to The Hospital for Sick Children, Toronto, Canada, through the SickKids Caribbean Initiative, for ancillary studies. IHC studies reported tumour cells positive for pankeratin, EMA, WT1 (C19) and vimentin with focal, dot-like positivity for desmin, partial mild staining for CD99 and focal staining for NSE. The desmoplastic stroma stained positive for SMA. There was intact nuclear staining with BAF47. Tumour cells were negative for WT1 (NH2), PAX 8, inhibin, glypican-3, CDX2, CK5/6, CK20, calretinin, CD30, myogenin, Heppar-1, and CD45. Cytogenetic studies using fluorescence *in situ* hybridization detected EWSR1 gene rearrangement. The final diagnosis was, therefore, a desmoplastic small round cell tumour.

## Discussion

**Figure 4.**
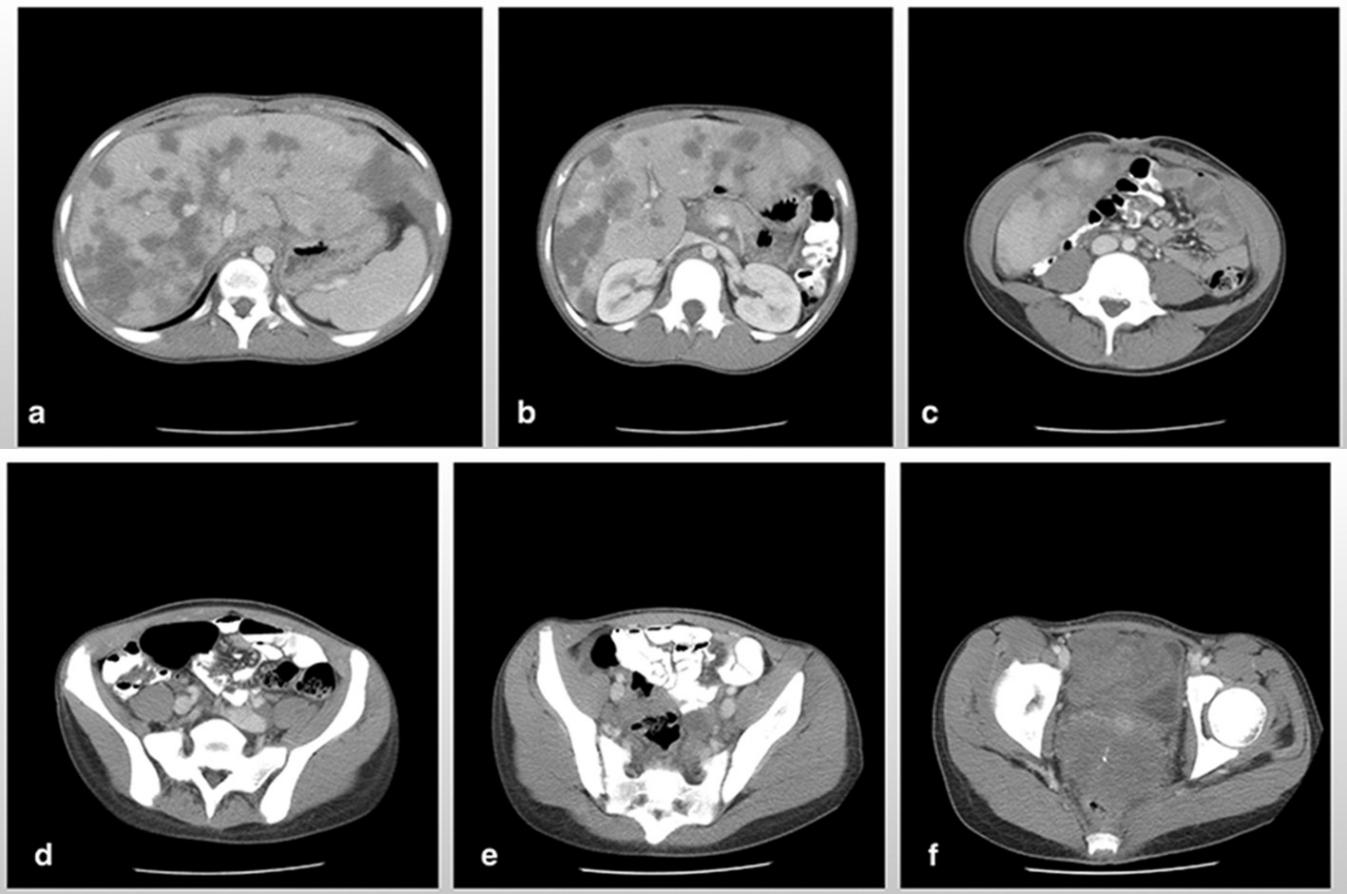
Axial CT scan of the abdomen and pelvis post-chemotherapy shows a significant reduction in the size of the hepatic lesions (a), resolution of hepatic vein and left renal vein stenosis from adjacent mass effect (b) and complete resolution of ascitic fluid (c). The previously reported midline and right adnexal masses were no longer seen (d) and (e) respectively. The large pelvic mass also demonstrated a significant reduction in size and extent (f).

**Figure 5.**
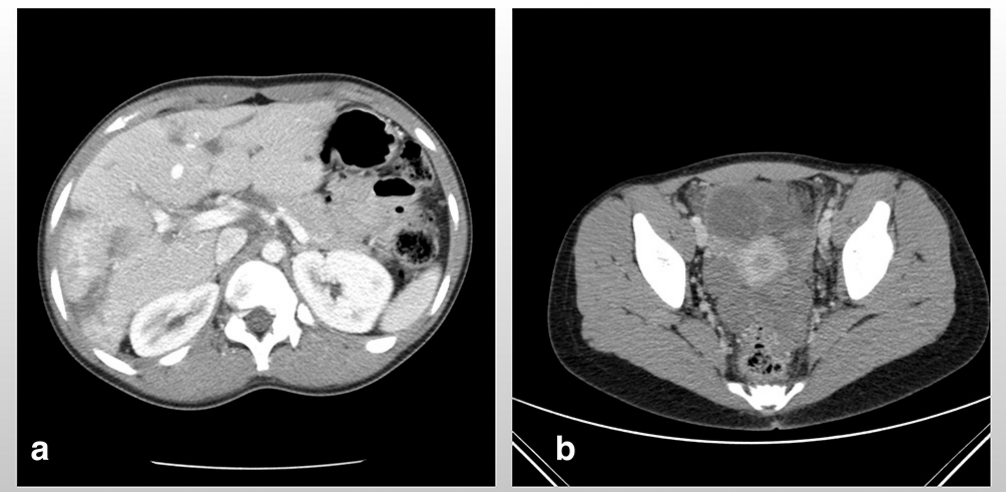
Axial CT scan of the abdomen and pelvis done approximately 1 year after 10 cycles of chemotherapy demonstrated a significant resolution of the hepatic lesions with residual low attenuation linear foci consistent with scarring and fibrosis (a). The pelvic mass showed a further significant reduction in size along with extensive low attenuation changes in keeping with necrosis. The uterus is now clearly visible surrounded by this pelvic mass (b).

Desmoplastic small round cell tumour (DSRCT) is a rare undifferentiated tumour associated primarily with serosal surfaces, particularly the peritoneum. Historically, this tumour demonstrates a predilection for adolescent males, with a typically aggressive course.^1^ One of the largest intra-abdominal DSRCT case series of 19 cases, Gerald et al, reported that the male to female ratio is greater than 5:1 and the age of presentation is approximately 18.6 years.^2^ The mean survival time was found to be approximately 29 months, with the longest recorded survivor up until the published date of the study is 101 months (approximately 8.5 years).^3^ The index case is female; no data or case was found suggesting differences in the course of the disease between the sexes. Most of the literature characterizing this tumour, however, was written in the late 20th century, with more recent studies emphasizing modern treatment modalities and their efficacy. There is a paucity of data outlining the variable clinical presentation of DSRCT. Less than 200 cases have been documented in the literature worldwide.^4^ Based on our review of the literature, to our knowledge, this is the first clinically as well as radiologically documented and published case of DSRCT in the Afro-Caribbean population. Abdominal pain, increased abdominal girth, and a palpable abdominal mass are identified as the first and most common symptoms experienced by affected patients.^3^ Additionally, obstipation, nausea, vomiting, anorexia, and weight loss are common presenting complaints.^5^ The index case presented with altered mentation, reduced bulk, increasing abdominal girth and firm non-tender hepatomegaly, not dissimilar to the clinical findings cited by other studies and case reports. It is possible that our patient’s altered mentation was due to hepatic encephalopathy; the liver function tests were normal except for elevated alkaline phosphatase and gamma-glutamyl transferase. The CT scan of the brain was normal. Fundamentally, the presentation of this tumour is highly non-specific, with histopathology and IHC the diagnostic methods of choice after identification with imaging. The observed literature reveals a modest benefit for the use of radiological investigations in the diagnosis of DSRCT. Published ultrasound images representative of this disease process were found to be almost non-existent during our literature search. In a study conducted by Gerald et al, the classic sonographic features were lobulated peritoneal masses, ascites, serosal hepatic metastasis and a thickened peritoneum.^1,6,7^ In another study by Bellah et al, CT was used to further characterize the radiological features of DSRCT, which included bulky intra-abdominal soft tissue masses involving omental and serosal surfaces, without any distinct organ of origin. Solid dominant heterogeneous pelvic masses in the retrovesical or rectouterine spaces, as well as concurrent metastases, which were common at the time of diagnosis were also reported, principally those involving lymph nodes and liver parenchyma.^6^ The CT scan of the abdomen and pelvis done for the indexed case demonstrated a large pelvic mass with extensive peritoneal deposits, nodal disease, hepatic metastases and ascites, largely similar to the findings described in the aforesaid study. The study investigation by W. Zhang et al found that *T*_1_ weighted magnetic resonance images demonstrated mild heterogeneous enhancement of the lesions in the aforementioned sites, with *T*_2_ weighted images showing mixed iso- and hyperintense areas. Positron emission tomography demonstrated multiple nodular foci of increased metabolic activity within the abdominopelvic masses.^7^ These studies highlight the relatively nonspecific features of DSRCT on imaging, but strongly suggest that the diagnosis should be suspected in young patients with multiple bulky heterogeneous peritoneal soft tissue masses, as well as suggest the use of imaging for staging and to guide biopsy. The differential diagnosis for these imaging findings included other small round blue cell tumours, namely rhabdomyosarcoma, malignant lymphoma, neuroblastoma along with atypical malignant mesothelioma. Gerald et al in 1991 described DSCRT as a characteristic lesion, when it was discovered that DSCRT has specific immunohistochemical and karyotypic features from other round blue cell tumours. Before 1991, DSCRT was considered an atypical presentation of small round blue cell tumours.^8^ ESWR1 gene rearrangement was detected via IHC of the histopathology sample taken from an ultrasound-guided liver biopsy of the indexed case. Interestingly, this particular phenomenon was found to be the most common finding elucidated in a large institutional comparative series by Mohamed et al of IHC techniques to diagnose DSRCT, with 91.3% of the tested samples in the study showing ESWR1 rearrangement using the fluorescent *in situ* hybridization technique, and 92.8% with the reverse transcription-polymerase chain reaction technique.^9^ These figures are consistent with the other existing literature exploring the cytogenetic diagnosis of DSRCT. Ultimately, despite aggressive multimodal therapy in combination with surgery, the prognosis of DSRCT remains very poor. Radiological investigations remain paramount in contextualizing the extent of organ involvement as well as facilitating biopsy to confirm this pathological entity. Multiplanar imaging is also critical in the assessment of the disease response to treatment. This patient was subsequently referred to the haematology and oncology service for chemotherapy where she was started on etoposide 155 mg, vincristine 2 mg, ifosfamide 2790 mg, and doxorubicin 116 mg intravenously. A CT scan of the chest, abdomen, and pelvis was done at 4 months after the first imaging was done which coincided with the completion of four cycles of chemotherapy to assess treatment response. The images of the chest were unremarkable. A significant response to treatment was observed in the CT scan of the abdomen and pelvis. Treatment strategy for DSRCT may also include radiation therapy, surgical resection of the tumour with peritonectomy and heated intraperitoneal chemotherapy. These treatment options were differed in this case. The images below depict a significant response to treatment 1 year after diagnosis post 10 cycles of chemotherapy (Figure 4; Figure 5).

This response though early in treatment appears promising for this patient. More data are needed to properly qualify the incidence of this condition in different population subtypes, to ascertain any genetic predisposition and/or other potential risk factors; environmental or otherwise that may lead to the development of DSRCT.

## Learning points

- DSRCT can mimic many other abdominopelvic malignancies and cannot be diagnosed on radiological imaging only. Histopathology and immunohistochemistry are essential in making the diagnosis.
- Whilst a useful initial investigation, abdominopelvic ultrasound is insufficient as it requires thorough characterization of the extent of the intra-abdominal and pelvic involvement of the tumour.
- The presentation of DSRCT is fairly consistent, but non-specific, with increasing abdominal girth in a young patient being a constant feature.
- DSRCT shows a strong predilection for the liver and peritoneum, relative to the other intra-abdominal and pelvic serosal organs.
